# Supplementary material for: Single-cell gene fusion detection by scFusion
Source: Nat Commun. 2022 Feb 28;13:1084. doi: 10.1038/s41467-022-28661-6 (PMC8885711; doi:10.1038/s41467-022-28661-6)
Supplement: Supplementary file 1 — Supplementary Information [file 41467_2022_28661_MOESM1_ESM.pdf]

Supplementary Information for

Single cell gene fusion detection by scFusion

Zijie Jin[1]; Wenjian Huang[2]; Ning Shen[3, 4]; Juan Li [5]; Xiaochen Wang[1]; Jiqiao Dong[6]; Peter J. Park[4]; Ruibin Xi [1,7,*].

1. School of Mathematical Sciences, Peking University, Beijing, 100871, China;
2. Academy for Advanced Interdisciplinary Studies, Peking University, Beijing, 100871, China;
3. Liangzhu Laboratory, Zhejiang University Medical Center, Hangzhou, Zhejiang Province, 311121, China;
4. Department of Biomedical Informatics, Harvard Medical School, Boston, MA, 02115, USA;
5. Department of Biomedical Engineering, College of Engineering, Peking University, Beijing, 100871, China;
6. GeneX Health Co. Ltd., Beijing, 100195, China.
7. Center for Statistical Science, Peking University, Beijing,100871, China.

* To whom correspondence should be addressed. E-mail: ruibinxi@math.pku.edu.cn

This PDF file includes:

Supplementary text
Supplementary Figure 1 to 14
SI References

Other supplementary materials for this manuscript include the following:

Supplementary Data 1 to 34

Supplementary Information Text

The convolutional neural network model

The convolutional neural network (CNN) model is mainly composed of three layers: a convolution layer, a pooling layer, and a fully connected layer (Supplementary Fig. 13a). The input of the network is a 60bp read, which contains the bases near the breakpoint of the fusion gene, and an integer between 15 and 45 that indicates the position of the breakpoint. The output is a probability that the sequence is a technical chimeric artefact.

We first convert the input sequence to a padded 5-row array that each row represents one of four bases (A, G, T, C) or the breakpoint position. Specifically, given the size of convolution kernel m, we convert a sequence S of length n to a $5 * (n + 2m - 2)$ array A in this way:

$$A_{i,j} = \begin{cases} 0.25 & \text{if } i = 1 \dots 4 \text{ and } j < m \text{ or } j > n - m \\ 1 & \text{if } (i, S_{j-m+1}) = (1, A) \text{ or } (2, G) \text{ or } (3, C) \text{ or } (4, T) \\ 1 & \text{if } i = 5 \text{ and the breakpoint position is } j - m + 1 \\ 0 & \text{otherwise} \end{cases} \quad (1)$$

where $A_{i,j}$ denotes the (i, j) element of A and S_i denotes the i-th base of S.

The convolutional layer takes the array as input, and each convolution kernel performs one-dimensional convolution on this 5-channel input. The number of kernels, as well as their size, are tunable parameters. In our model, we use 2048 convolution kernels of length 16 ($m = 16$) by default. Therefore, the output of this layer is of size 2048*75. The output feature map of each kernel is then rectified by adding a bias and being activated by ReLU.

The pooling layer performs the max operation on the rectified features, aiming to integrate the features obtained by the convolution. After this stage, we get a 2048-dimensional vector. Finally, the feature vector is passed to the fully connected layer, which consists of a hidden layer and an output layer. The hidden layer consists of 256 neurons by default and the output layer is a single neuron with the sigmoid function.

We use negative log-likelihood as the loss function and Adam[1] with L2 regularization for optimization. We use mini-batch to speed up training and add a dropout layer after convolution to reduce overfitting. By default, batch size, learning rate, weight decay, and the dropout rate is set to 512, $3*10^{-3}$, $1*10^{-4}$, and 0.25 respectively.

Commands

We list how we use the bulk method in the following.

STAR mapping:

STAR --runThreadN 20 --genomeDir CTAT_GENOME_LIB/GRCh37_ gencode_v19_CTAT_lib_Feb092018/ctat_genome_lib_ build_dir/ref_genome.fa.star.idx --readFilesIn /1_1.fastq 1_2.fastq --outSAMtype BAM SortedByCoordinate --chimOutType SeparateSAMold --outSAMunmapped Within KeepPairs --quantMode GeneCounts --outFileNamePrefix 1/human --chimSegmentMin 12 --chimJunctionOverhangMin 12 --alignSJDBoverhangMin 10 --alignMatesGapMax 100000 --alignIntronMax 100000 --chimSegmentReadGapMax 3 --alignSJstitchMismatchNmax 5 -1 5 5

STAR-Fusion:

STAR-Fusion-v1.8.1/STAR-Fusion --min_novel_junction_support 3 --min_sum_frags 3 --left_fq 1_1.fastq --right_fq 1_2.fastq --genome_lib_dir /GRCh37_gencode_v19_CTAT_lib_Oct012019.plug-n-play/ctat_genome_lib_build_dir/ --CPU 20 --output_dir STAR_Fusion_Res/

EricScript:

Ericscript/ericscript.pl -db Ericscript/ericscript_db_homosapiens_ensembl73 -p 20 -name 1 -o EricRes/1 1_1.fastq 1_2.fastq

Arriba:

Arriba is piped with STAR:

STAR --runThreadN 20 --genomeDir CTAT_GENOME_LIB/GRCh37_ gencode_v19_CTAT_lib_Feb092018/ctat_genome_lib_ build_dir/ref_genome.fa.star.idx --genomeLoad NoSharedMemory --readFilesIn 1_1.fastq 1_2.fastq --outStd BAM_Unsorted --outFileNamePrefix OUTDIR --outSAMtype BAM Unsorted --outSAMunmapped Within --outBAMcompression 0 --outFilterMultimapNmax 1 --outFilterMismatchNmax 3 --chimSegmentMin 10 --chimOutType WithinBAM SoftClip --chimJunctionOverhangMin 10 --chimScoreMin 1 --chimScoreDropMax 30 --chimScoreJunctionNonGTAG 0 --chimScoreSeparation 1 --alignSJstitchMismatchNmax 5 -1 5 5 --chimSegmentReadGapMax 3 | arriba -x /dev/stdin -o OUTDIR/fusions.tsv -O OUTDIR/fusions.discarded.tsv -a "$ASSEMBLY_FA" -g "$ANNOTATION_GTF" -b "$BLACKLIST_TSV" -T -P -S 3

FusionCatcher:

FusionCatcher/bin/fusioncatcher -d FusionCatcher/data/human_v95 -i 1 1.fastq,1_2.fastq -o FusionCatcherRes

Figures

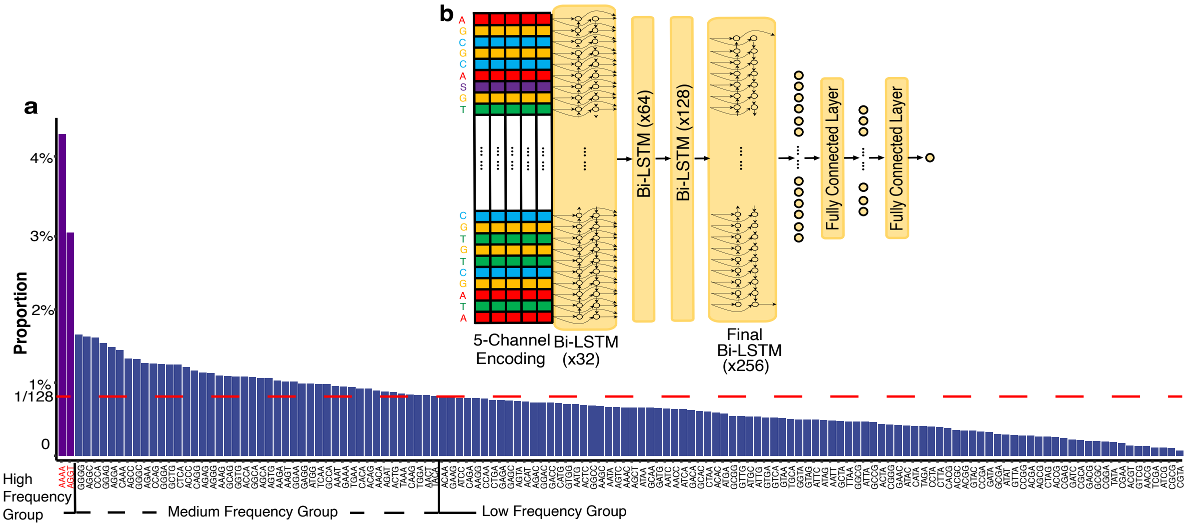

Supplementary Fig. 1. The bi-LSTM model. (**a**) The occurrence frequencies of the 128 possible 4-bp sub-sequences around the junction of chimeric reads. The red dashed line is the expected occurrence frequency if the 4bp sequence occurred randomly. (**b**) The design of the bi-LSTM. Source data are provided as a Source Data file.

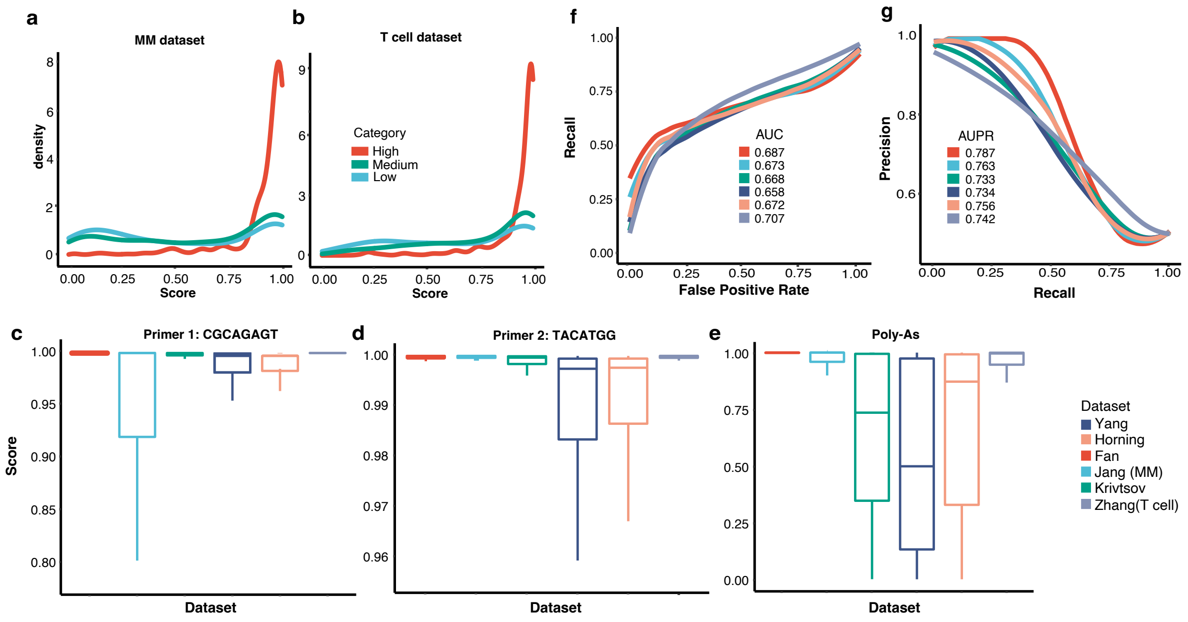

Supplementary Fig. 2. The performance of bi-LSTM model. (**a, b**) The distributions of artefact scores of chimeric reads in the high-, middle-, and low- frequency group. The high-frequency group consists of chimeric reads with AAAA and AGGT as their junction sequences (frequency > 3%). The low-frequency group consists of chimeric reads whose 4bp junction sequences occurred in less than 1/128 of all chimeric reads. The medium frequency group is the other chimeric reads. (**a**) The MM dataset. (**b**) The T cell data. (**c, d, e**) The boxplots of artefact scores of reads containing (**c**) CGCAGAGT (n=113132, 13382, 141592, 81613, 109470, and 6384 reads, respectively), (**d**) TACATGG (n=47552, 7335, 86613, 48426, 55794, and 9542 reads, respectively), and (**e**) poly-As in six datasets (n=213562, 4286, 60912, 3744, 9717, and 5304 reads, respectively). The center line in the boxplot is the median, bounds of boxes are the interquartile of the data, whiskers represent minima/maxima excluding outliers and dots represents outliers of beyond 1.5*Interquartile Range (IQR) from either end of the box. The sequences CGCAGAGT and TACATGG are often used as primer[2,3] sequences. These chimeric reads might be generated by random annealing of complementary sequences. (**f**)The ROCs and (**g**) PR curves of the Bi-LSTM without retraining. Source data are provided as a Source Data file.

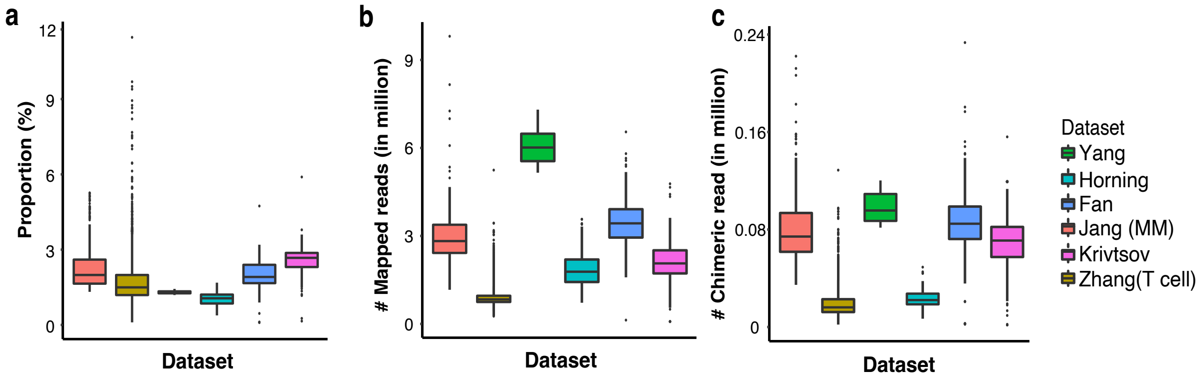

Supplementary Fig. 3. Statistics of chimeric reads. The boxplots of (**a**) the proportions of chimeric reads in all mapped reads, (**b**) the total number of mapped reads in different scRNA-seq data and (**c**) the total number of chimeric reads (n=21, 147, 173, 449, 215, and 7,069 cells, respectively). The center line in the boxplot is the median, bounds of boxes are the interquartile of the data, whiskers represent minima/maxima excluding outliers and dots represents outliers of beyond 1.5*Interquartile Range (IQR) from either end of the box. Source data are provided as a Source Data file.

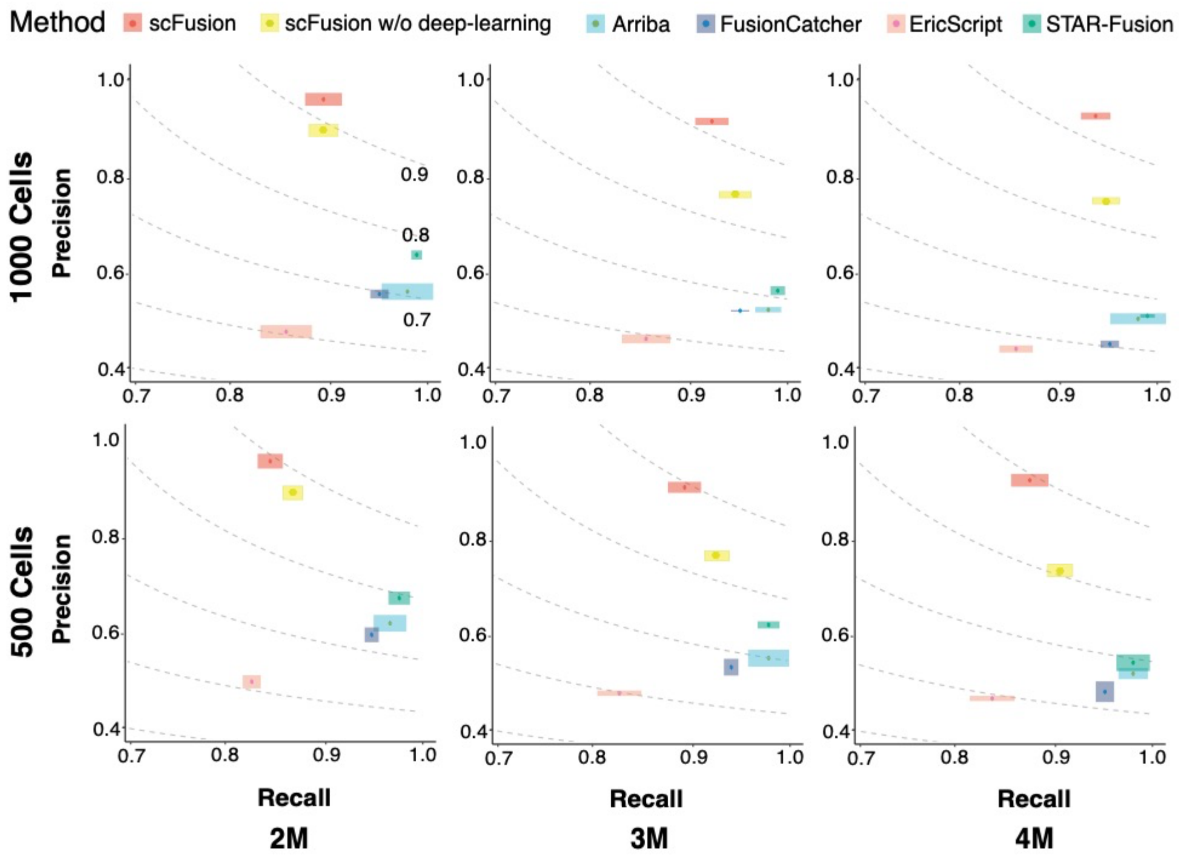

Supplementary Fig. 4. The precisions and recalls of scFusion, scFusion without deep-learning model, and four bulk methods in six different simulation setups. The figures in the two rows correspond to simulations with 1,000 cells and 500 cells, and the figures in the three columns correspond to simulations with 2 million, 3 million, and 4 million reads in each data. The dots in the figures are the means of precisions and recalls of ten simulations in each setup and the boxes ±1 standard deviation (SD) of the precisions and recalls (n=10 simulations). The dashed lines are the contour lines with constant F-scores (F-scores are marked in the top-left figure).

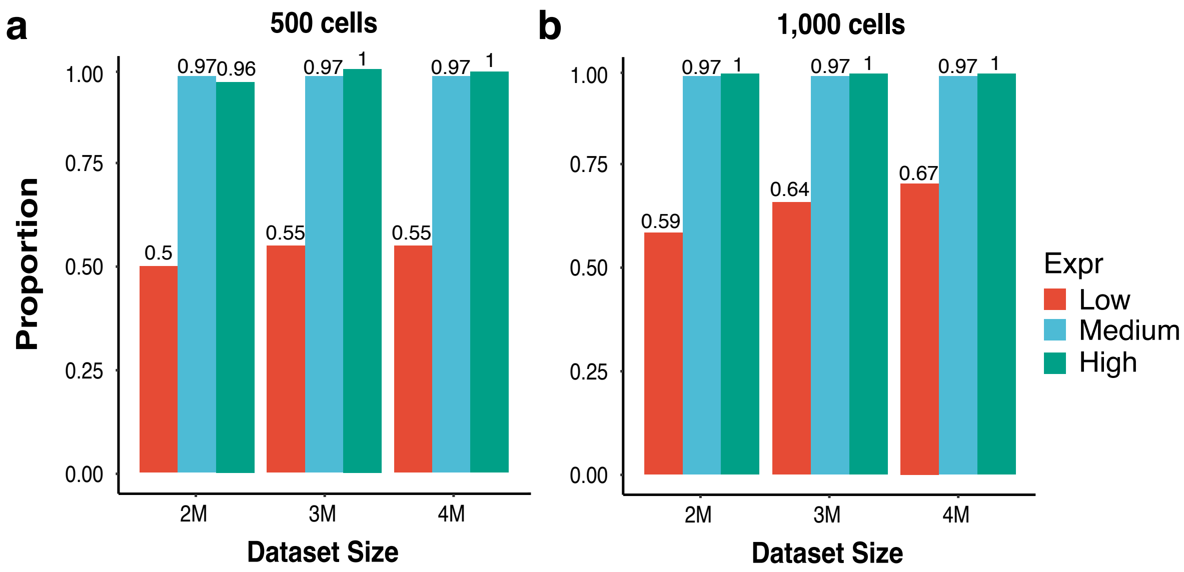

Supplementary Fig. 5. The detection powers of fusions at different expression levels. The detection powers of fusions at different expression levels using (**a**) 500 and (**b**) 1000 cells.

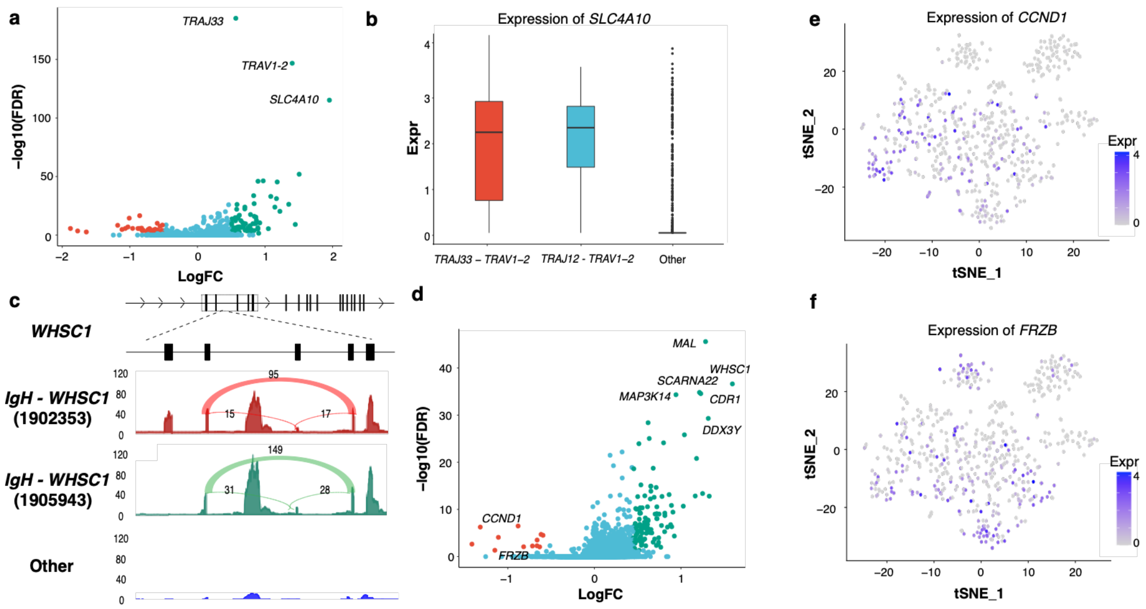

Supplementary Fig. 6. Fusions are correlated with gene up-regulation. (**a**) The volcano plots of the differential expression analysis of T cells between cells with and without the *TRAJ33-TRAV1-2* or *TRAJ12-TRAV1-2* recombination. (**b**) The *SLC4A10* expression is significantly higher in cells with the *TRAJ33-TRAV1-2* or *TRAJ12-TRAV1*-2 recombination than in other cells (n=2,355 cells). The center line in the boxplot is the median, bounds of boxes are the interquartile of the data, whiskers represent minima/maxima excluding outliers and dots represents outliers of beyond 1.5*Interquartile Range (IQR) from either end of the box. (**c**) The local view of the mean read depth of *WHSC1* at different locations for the cells with the two *IgH-WHSC1* fusions and the cells without the fusions. (**d**) The volcano plots of the differential expression genes between cells with and without the *IgH-WHSC1* fusion. (**e**) The expression of *CCND1* shown in the tSNE plot. (**f**) The expression of *FRZB* shown in the tSNE plot. Source data are provided as a Source Data file.

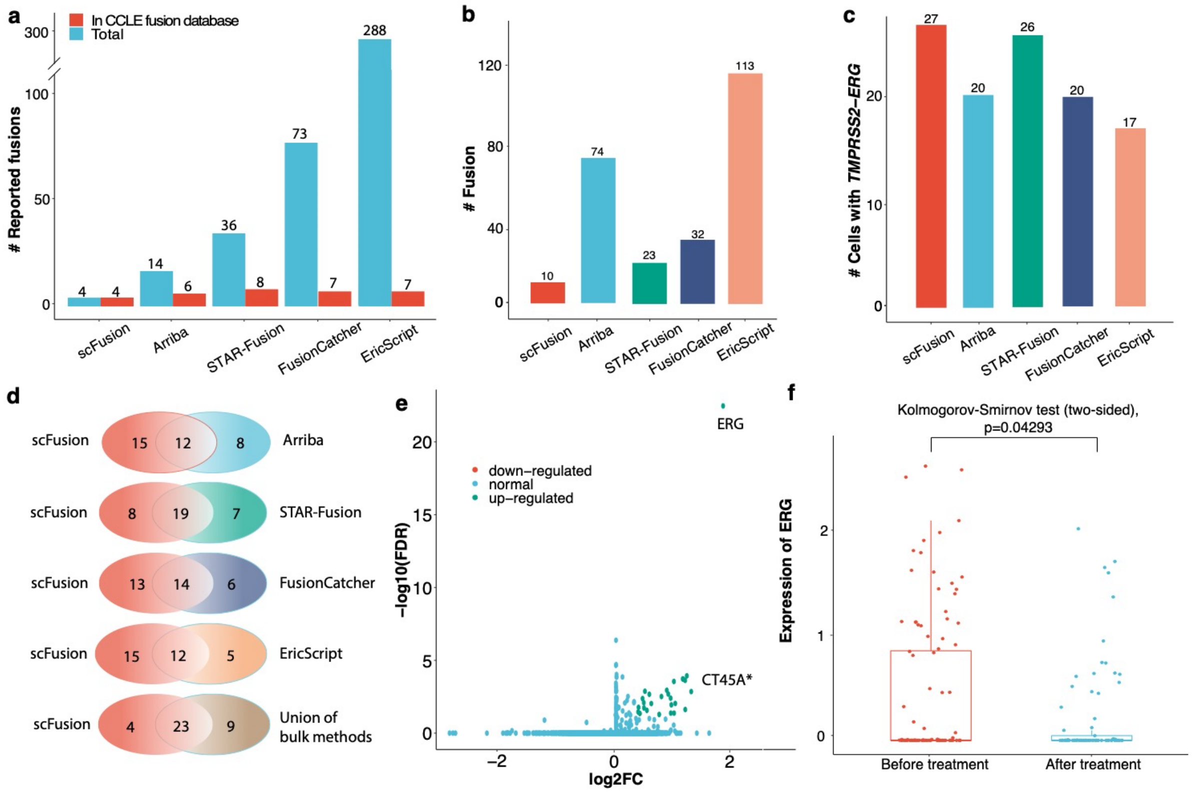

Supplementary Fig. 7. The prostate data. (**a**) The number of detected gene fusions by five methods in the prostate LNCaP data. (**b**) The number of detected gene fusions by five methods in the prostate patient data. (**c**) The number of cells with the *TMPRSS2-ERG* fusion by five methods. (**d**) The Venn plot of the number of cells harboring the *TMPRSS2-ERG* fusion. (**e**) The volcano plot of the differential expression genes of cells between cells with and without the *TMPRSS2-ERG* fusion. (**f**) The expression of ERG before and after the treatment (n=425 cells). The center line in the boxplot is the median, bounds of boxes are the interquartile of the data, whiskers represent minima/maxima excluding outliers and dots represents outliers of beyond 1.5*Interquartile Range (IQR) from either end of the box. Source data are provided as a Source Data file.

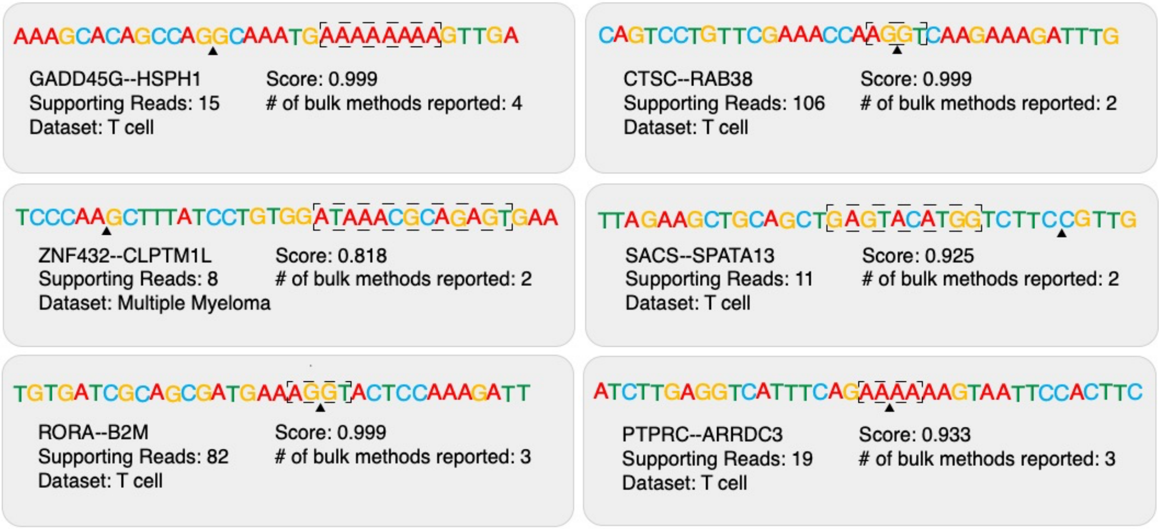

Supplementary Fig. 8. Six examples of gene fusions with high artefact scores. The black triangles show the junctions of gene fusions. The patterns in the dashed boxes might explain their high artefact scores.

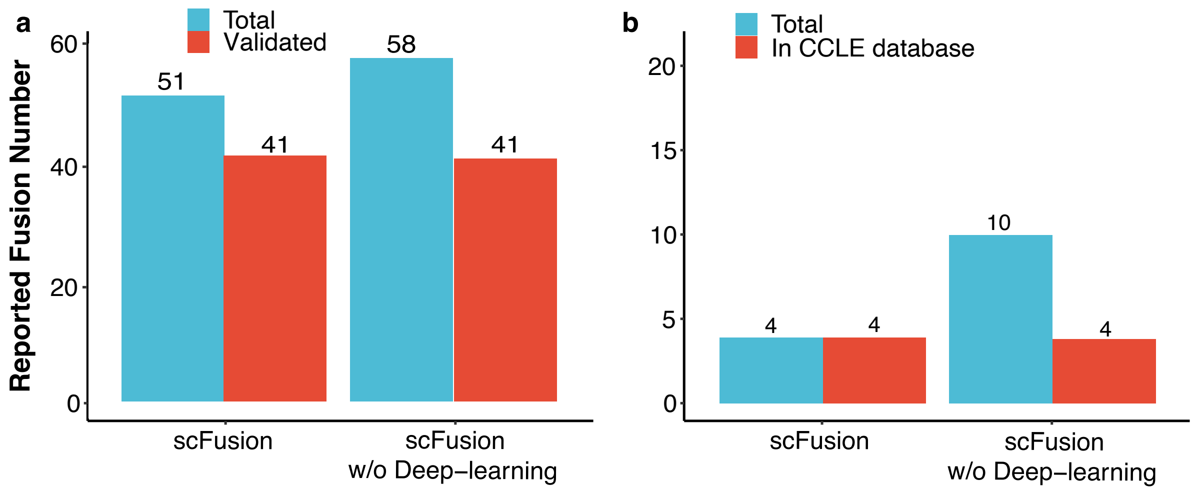

Supplementary Fig. 9. Number of fusion candidates reported by scFusion when turning off the deep-learning model in (a) the spike-in data and (b) the LNCaP data. In the figure, "Total" means the total number of reported fusions. "Validated" means the number of fusions in the 27 spiked-in fusions or supported by bulk reads. "In CCLE database" means the number of reported fusions listed in the CCLE fusion database of the LNCaP cell line.

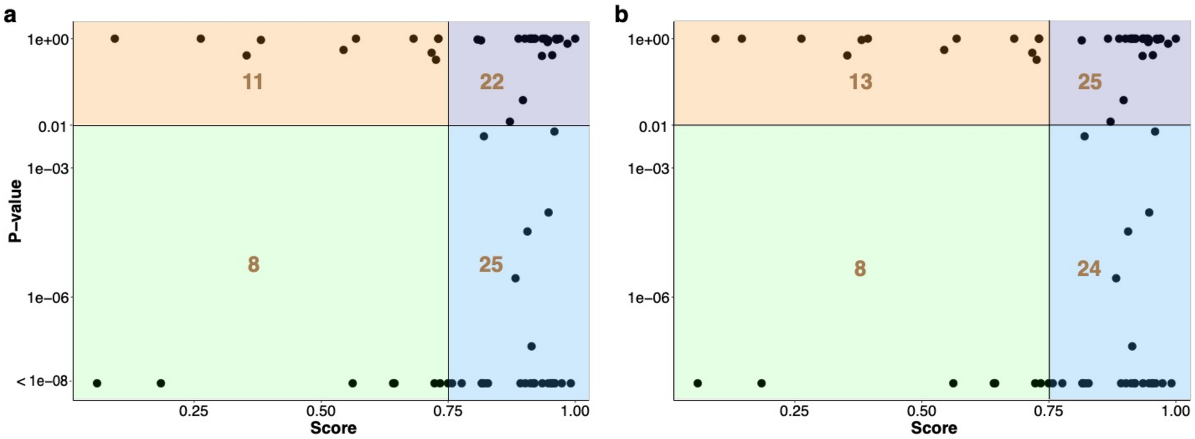

Supplementary Fig. 10. The scatterplot of the artefact scores against p-values for the false positives reported by (a) STAR-Fusion and (b) Arriba. The numbers in the figure are the numbers of points in the four different regions. If we set the p-value cutoff as 0.01, 25 and 24 of the false positives predicted by STAR-Fusion and Arriba had significant p-values (<0.01) and thus cannot be filtered by the p-values but can be filtered by the deep learning model due to their large artefact scores (>0.75). Note that this analysis focuses on false positives reported by the bulk methods. Many false positives have been filtered by the build-in filters in the bulk methods or by the ≥ 2 cell filter. Hence, the deep learning model should be more effective than in this figure. Source data are provided as a Source Data file.

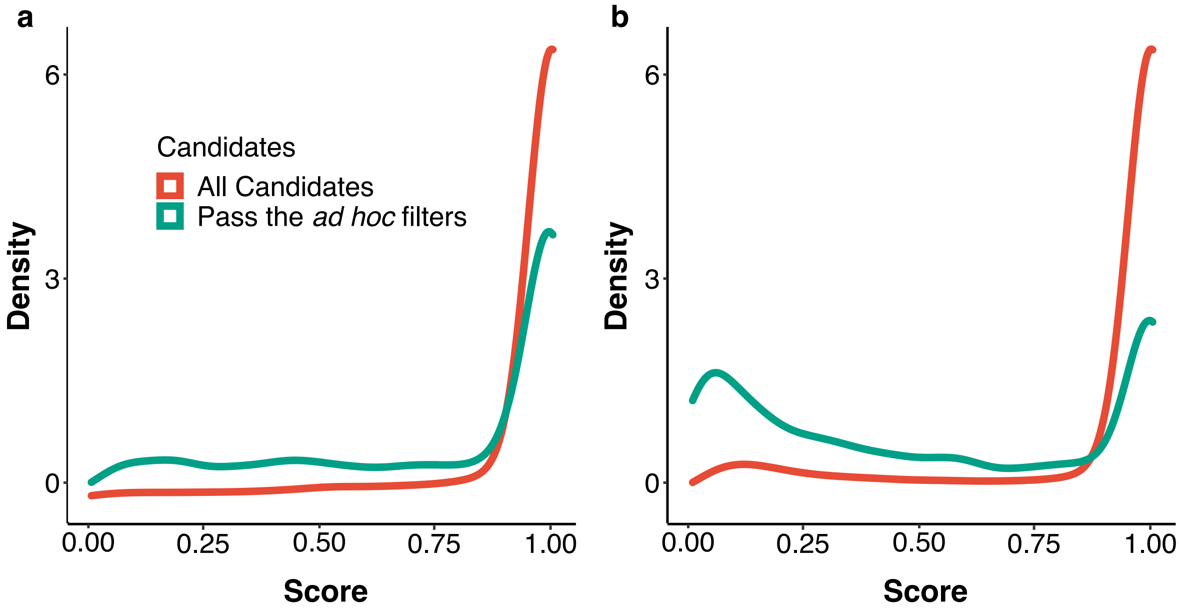

Supplementary Fig. 11. The density plots of scores of fusion candidates from (a) LNCaP data and (b) prostate patient data. The red curves are the densities of all candidates from that data, and the blue and green curves are the densities of candidates passed the first filter and the second filter in Figure 4b, respectively. Source data are provided as a Source Data file.

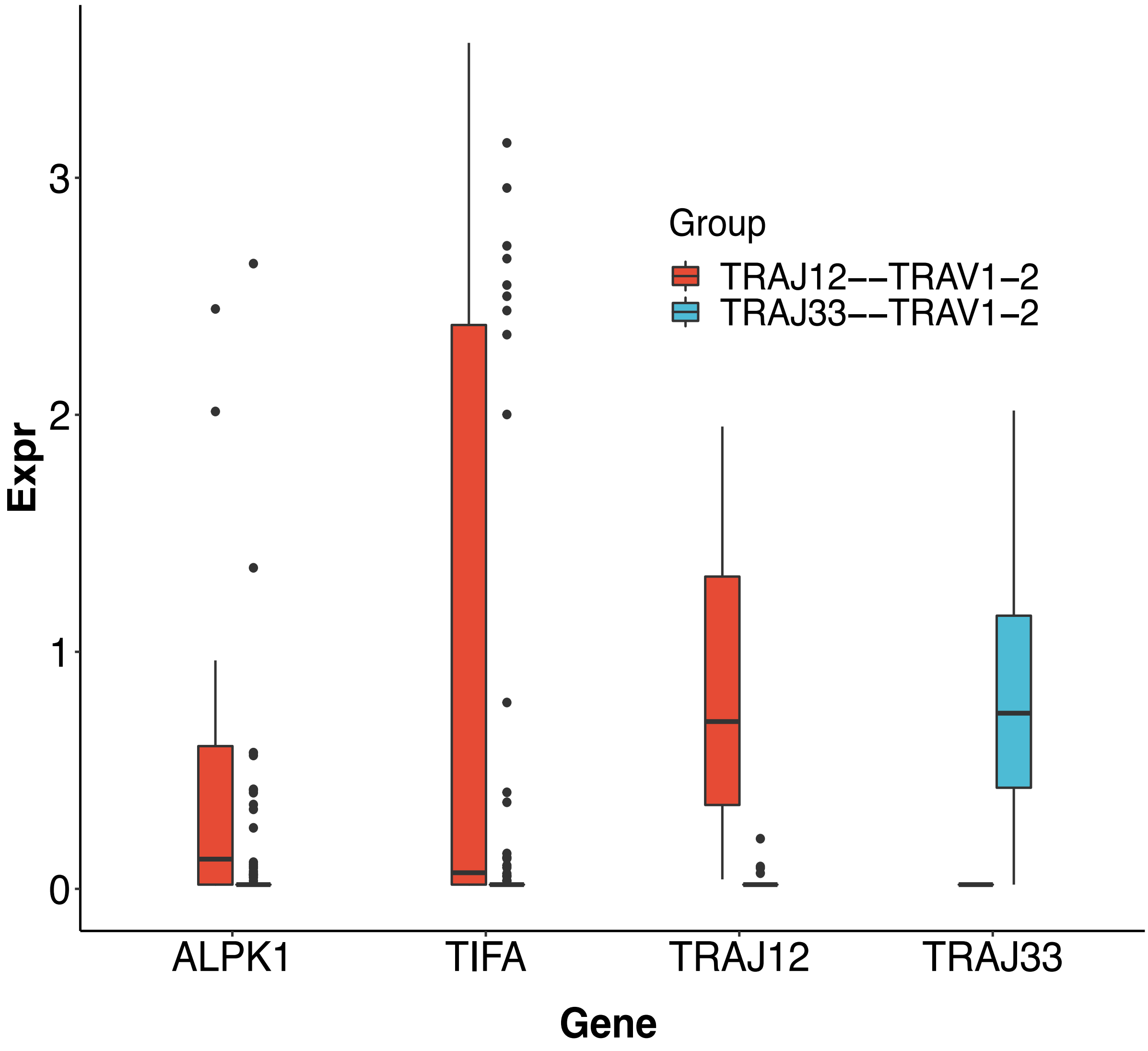

Supplementary Fig. 12. Differential expression analysis. The expression of *ALPK1*, *TIFA, TRAJ33* and *TRAJ12* in the two cell groups (n=592 cells). The center line in the boxplot is the median, bounds of boxes are the interquartile of the data, whiskers represent minima/maxima excluding outliers and dots represents outliers of beyond 1.5*Interquartile Range (IQR) from either end of the box. Source data are provided as a Source Data file.

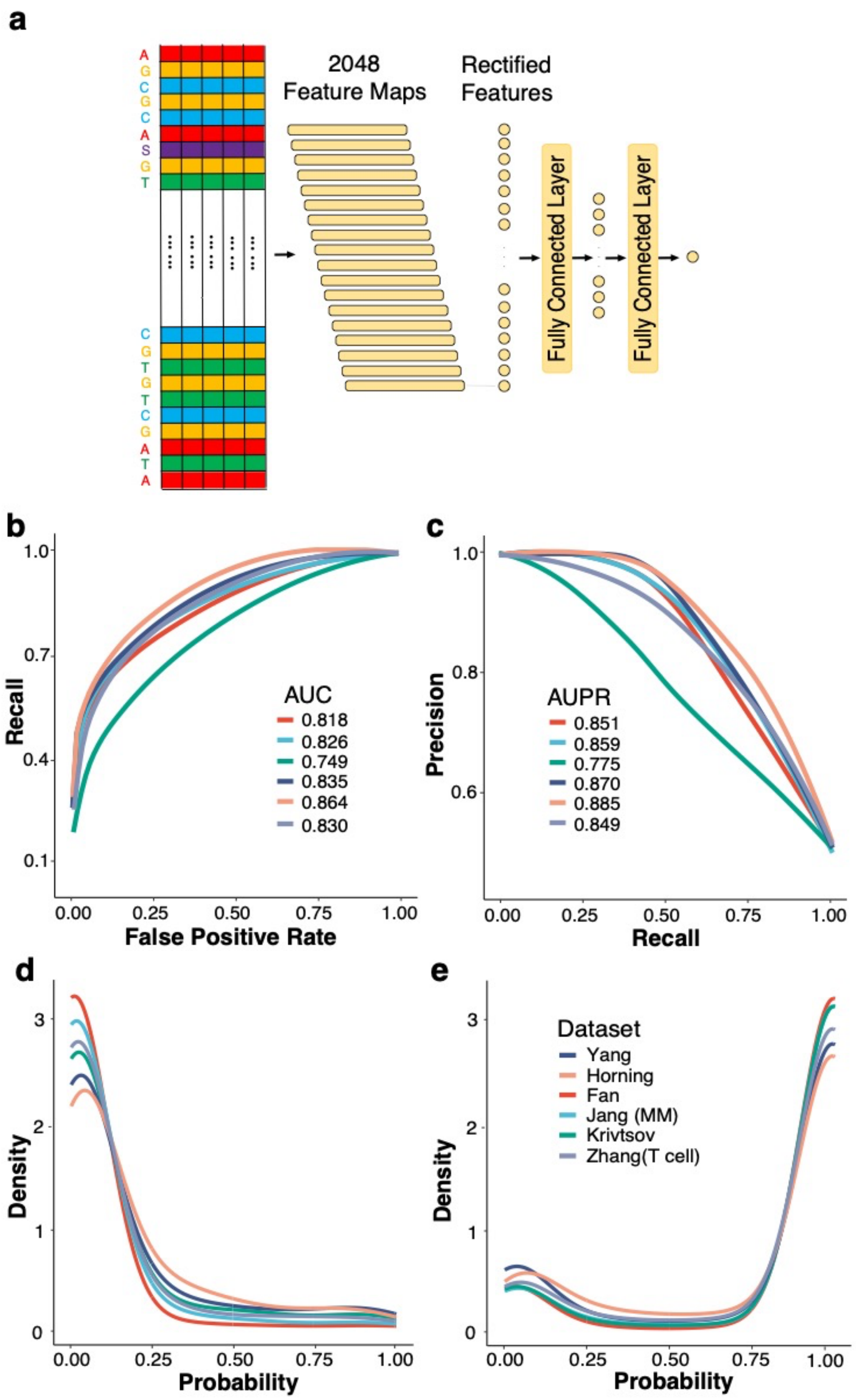

Supplementary Fig. 13. The CNN model. (**a**) The design of the CNN model (**b**) The ROCs of the CNN model for different single-cell datasets. The AUCs are also shown. (**c**) The PR curves and their AUPRs of the CNN model. (**d**) The densities of the technical artefact score of gene fusions in the PCAWG study by the CNN models retrained using six different datasets. (**e**) The densities of the technical artefact score of chimeric reads. The models are retrained using different datasets. Source data are provided as a Source Data file.

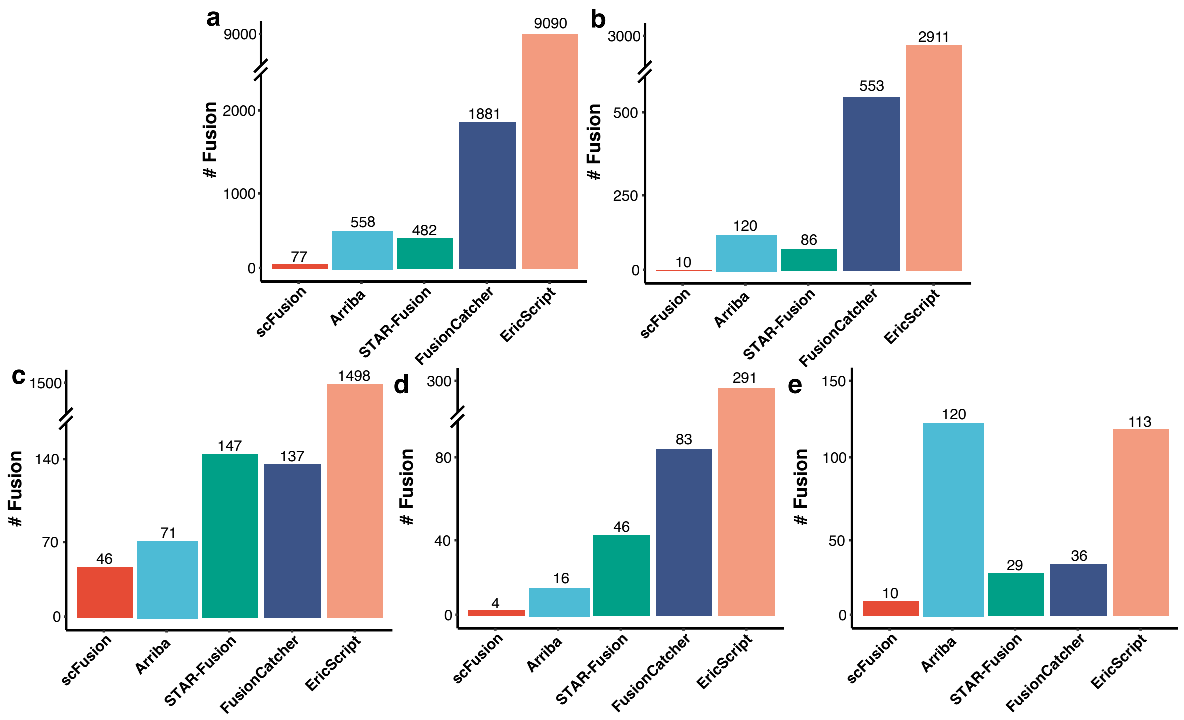

Supplementary Fig. 14. The numbers of reported gene fusions if the no-approved-symbol and lncRNA- filters are not applied in (a) Spike-in, (b) T-cell, (c) MM, (d) LNCaP, and (e) prostate patient data.

Reference

1 Kingma, D. P. & Ba, J. Adam: A method for stochastic optimization. *arXiv preprint arXiv:1412.6980*, (2014).

2 Yamaguchi, S. *et al.* Cell Dynamics and Expression of Tumor Necrosis Factor (TNF)-α, Interleukin-6, and TNF Receptors in Angioimmunoblastic Lymphadenopathy-Type T Cell Lymphoma. *Experimental and Molecular Pathology* **68**, 85-94, (2000).

3 Lu, J. *et al.* A novel serine protease inhibitor from Bungarus fasciatus venom. *Peptides* **29**, 369-374, (2008).
