# Supplementary material for: Single-cell gene fusion detection by scFusion
Source: Nat Commun. 2022 Feb 28;13:1084. doi: 10.1038/s41467-022-28661-6 (PMC8885711; doi:10.1038/s41467-022-28661-6)
Supplement: Supplementary file 2 — Reporting Summary [file 41467_2022_28661_MOESM2_ESM.pdf]

nature portfolio

Corresponding author(s): Ruibin Xi

Last updated by author(s): Jan 12, 2022

Reporting Summary

Nature Portfolio wishes to improve the reproducibility of the work that we publish. This form provides structure for consistency and transparency in reporting. For further information on Nature Portfolio policies, see our Editorial Policies and the Editorial Policy Checklist.

Statistics

For all statistical analyses, confirm that the following items are present in the figure legend, table legend, main text, or Methods section.

n/a	Confirmed	
☐	☒	The exact sample size (*n*) for each experimental group/condition, given as a discrete number and unit of measurement
☒	☐	A statement on whether measurements were taken from distinct samples or whether the same sample was measured repeatedly
☐	☒	The statistical test(s) used AND whether they are one- or two-sided<br>*Only common tests should be described solely by name; describe more complex techniques in the Methods section.*
☒	☐	A description of all covariates tested
☐	☒	A description of any assumptions or corrections, such as tests of normality and adjustment for multiple comparisons
☐	☒	A full description of the statistical parameters including central tendency (e.g. means) or other basic estimates (e.g. regression coefficient) AND variation (e.g. standard deviation) or associated estimates of uncertainty (e.g. confidence intervals)
☐	☒	For null hypothesis testing, the test statistic (e.g. *F*, *t*, *r*) with confidence intervals, effect sizes, degrees of freedom and *P* value noted<br>*Give P values as exact values whenever suitable.*
☒	☐	For Bayesian analysis, information on the choice of priors and Markov chain Monte Carlo settings
☒	☐	For hierarchical and complex designs, identification of the appropriate level for tests and full reporting of outcomes
☐	☒	Estimates of effect sizes (e.g. Cohen's *d*, Pearson's *r*), indicating how they were calculated

Our web collection on statistics for biologists contains articles on many of the points above.

Software and code

Policy information about availability of computer code

Data collection	No software was used.
Data analysis	FusionCatcher v1.10; Arriba v1.0.1; EricScript v0.5.5b; STAR-Fusion v1.8.1; STAR v2.7.4a; Seurat v3.2.0; FusionSimulatorToolkit (no version number); R v4.0.2; Python v3.7.9; Samtools v1.10; Bedtools v2.29.2. scFusion is available at GitHub (https://github.com/XiDsLab/scFusion).

For manuscripts utilizing custom algorithms or software that are central to the research but not yet described in published literature, software must be made available to editors and reviewers. We strongly encourage code deposition in a community repository (e.g. GitHub). See the Nature Portfolio guidelines for submitting code & software for further information.

Data

Policy information about availability of data

All manuscripts must include a data availability statement. This statement should provide the following information, where applicable:

- Accession codes, unique identifiers, or web links for publicly available datasets
- A description of any restrictions on data availability
- For clinical datasets or third party data, please ensure that the statement adheres to our policy

Raw data of the spike-in data generated in this study have been deposited in the Genome Sequence Archive (GSA) of BIG Data Center, Beijing Institute of Genomics (BIG), Chinese Academy of Sciences, with an accession number code HRA001199 (https://bigd.big.ac.cn/gsa-human/browse/HRA001199). The scRNA-seq data used in this study are available in Gene Expression Omnibus (GEO) under accession code GSE81812 (https://www.ncbi.nlm.nih.gov/geo/query/acc.cgi?acc=GSE81812), GSE99795 (https://www.ncbi.nlm.nih.gov/geo/query/acc.cgi?acc=GSE99795), GSE110499, (https://www.ncbi.nlm.nih.gov/geo/query/acc.cgi?acc=GSE110499), GSE118900 (https://www.ncbi.nlm.nih.gov/geo/query/acc.cgi?acc=GSE118900), GSE127298 (https://www.ncbi.nlm.nih.gov/geo/query/acc.cgi?acc=GSE127298), GSE140228 (https://www.ncbi.nlm.nih.gov/geo/query/acc.cgi?acc=GSE140228https://www.ncbi.nlm.nih.gov/geo/). Raw sequence data of the prostate patient data

used in this study are available in dbGaP under accession code(accession phs001988.v1.p1) (https://www.ncbi.nlm.nih.gov/projects/gap/cgi-bin/study.cgi?study_id=phs001988.v1.p1).

Field-specific reporting

Please select the one below that is the best fit for your research. If you are not sure, read the appropriate sections before making your selection.

- [x] Life sciences
- [ ] Behavioural & social sciences
- [ ] Ecological, evolutionary & environmental sciences

For a reference copy of the document with all sections, see nature.com/documents/nr-reporting-summary-flat.pdf

Life sciences study design

All studies must disclose on these points even when the disclosure is negative.

Sample size	No sample-size calculation was performed. The study is about a new computational method for analyzing scRNA-seq data. In this study, we spiked-in gene fusions to single cells and performed single cell RNA sequencing using SMART-seq2 to evaluate the fusion detection algorithm. Simulation showed that sensitive detection of gene fusions need ~ 20 cells for each fusion and we chose to spike-in 27 cells for each fusion. We chose to spiked-in 27 gene fusions for stable evaluation of the algorithms. This gave us 729 cells of scRNA-seq data, largely similar to the cell numbers in studies using full-length scRNA-seq techonologies (such as SMART-seq2) (1, 2).<br>Reference:<br>1. Zhang, Q. et al. Landscape and Dynamics of Single Immune Cells in Hepatocellular Carcinoma. Cell 179, 829-845.e820, (2019).<br>2. Jang, J. S. et al. Molecular signatures of multiple myeloma progression through single cell RNA-Seq. Blood Cancer Journal 9, 2, (2019).
Data exclusions	No data were excluded.
Replication	Not replicated. The study is about a new computational method and does not involve experimental findings.
Randomization	Not relevant. The study does not involve different experimental groups.
Blinding	Not relevant. No group allocation is involved in the study.

Reporting for specific materials, systems and methods

We require information from authors about some types of materials, experimental systems and methods used in many studies. Here, indicate whether each material, system or method listed is relevant to your study. If you are not sure if a list item applies to your research, read the appropriate section before selecting a response.

Materials & experimental systems

n/a	Involved in the study	
x		Antibodies
	x	Eukaryotic cell lines
x		Palaeontology and archaeology
x		Animals and other organisms
x		Human research participants
x		Clinical data
x		Dual use research of concern

Methods

n/a	Involved in the study	
x		ChIP-seq
x		Flow cytometry
x		MRI-based neuroimaging

Eukaryotic cell lines

Policy information about cell lines

Cell line source(s)	The 293T cells were purchased from the National Infrastructure of Cell Line Resource (http://www.cellresource.cn/).
Authentication	None of the cell lines used were authenticated.
Mycoplasma contamination	All cell lines tested negative for mycoplasma contamination.
Commonly misidentified lines (See ICLAC register)	no commonly misidentified cell lines were used in the study.
