# Supplementary material for: Single-cell gene fusion detection by scFusion
Source: Nat Commun. 2022 Feb 28;13:1084. doi: 10.1038/s41467-022-28661-6 (PMC8885711; doi:10.1038/s41467-022-28661-6)
Supplement: Supplementary file 4 — Description of Additional Supplementary Files [file 41467_2022_28661_MOESM4_ESM.pdf]

Title: Supplementary Data 1

Description: True gene fusions in simulation experiments.

Title: Supplementary Data 2

Description: Reported fusions by scFusion in one of the simulation data (the scenario with 1,000 cells and 4 M reads).

Title: Supplementary Data 3

Description: Reported fusions by Arriba in one of the simulation data (the scenario with 1,000 cells and 4 M reads)

Title: Supplementary Data 4

Description: Reported fusions by FusionCatcher in one of the simulation data (the scenario with 1,000 cells and 4 M reads)

Title: Supplementary Data 5

Description: Reported fusions by EricScript in one of the simulation data (the scenario with 1,000 cells and 4 M reads)

Title: Supplementary Data 6

Description: Reported fusions by STAR-Fusion in one of the simulation data (the scenario with 1,000 cells and 4 M reads)

Title: Supplementary Data 7

Description: Spiked-in fusions in our experiment.

Title: Supplementary Data 8

Description: Reported fusions by scFusion in the spike-in data.

Title: Supplementary Data 9

Description: Reported fusions by Arriba in the spike-in data.

Title: Supplementary Data 10

Description: Reported fusions by FusionCatcher in the spike-in data.

Title: Supplementary Data 11

Description: Reported fusions by EricScript in the spike-in data.

Title: Supplementary Data 12

Description: Reported fusions by STAR-Fusion in the spike-in data.

Title: Supplementary Data 13

Description: Reported fusions by scFusion in the T cell data.

Title: Supplementary Data 14

Description: Reported fusions by Arriba in the T cell data.

Title: Supplementary Data 15

Description: Reported fusions by FusionCatcher in the T cell data.

Title: Supplementary Data 16

Description: Reported fusions by EricScript in the T cell data.

Title: Supplementary Data 17

Description: Reported fusions by STAR-Fusion in the T cell data.

Title: Supplementary Data 18

Description: Reported fusions by scFusion in the multiple myeloma data.

Title: Supplementary Data 19

Description: Reported fusions by Arriba in the multiple myeloma data.

Title: Supplementary Data 20

Description: Reported fusions by FusionCatcher in the multiple myeloma data.

Title: Supplementary Data 21

Description: Reported fusions by EricScript in the multiple myeloma data.

Title: Supplementary Data 22

Description: Reported fusions by STAR-Fusion in the multiple myeloma data.

Title: Supplementary Data 23

Description: Differential expressed genes in the cells with *IgH-WHSC1* fusions. Two-sided Wilcoxon rank-sum test was used and p-values were adjusted by the Benjamini & Hochberg method.

Title: Supplementary Data 24

Description: Reported fusions by scFusion in the prostate LNCaP data.

Title: Supplementary Data 25

Description: Reported fusions by Arriba in the prostate LNCaP data.

Title: Supplementary Data 26

Description: Reported fusions by FusionCatcher in the prostate LNCaP data.

Title: Supplementary Data 27

Description: Reported fusions by EricScript in the prostate LNCaP data.

Title: Supplementary Data 28

Description: Reported fusions by STAR-Fusion in the prostate LNCaP data.

Title: Supplementary Data 29

Description: Reported fusions by scFusion in the prostate patient data.

Title: Supplementary Data 30

Description: Reported fusions by Arriba in the prostate patient data.

Title: Supplementary Data 31

Description: Reported fusions by FusionCatcher in the prostate patient data.

Title: Supplementary Data 32

Description: Reported fusions by EricScript in the prostate patient data.

Title: Supplementary Data 33

Description: Reported fusions by STAR-Fusion in the prostate patient data.

Title: Supplementary Data 34

Description: Differential expressed genes in the cells with *TRAJ33-TRAV1-2* fusions. Two-sided Wilcoxon rank-sum test was used and p-values were adjusted by the Benjamini & Hochberg method.
